# Postpartum Care Differences in LGBTQ+ and Non-LGBTQ+ Individuals

**DOI:** 10.1001/jamahealthforum.2025.0672

**Published:** 2025-05-02

**Authors:** Kevin H. Nguyen, Jamie R. Daw, Heidi L. Allen

**Affiliations:** 1 Department of Health Law, Policy, and Management, Boston University School of Public Health, Boston, Massachusetts; 2 Department of Health Policy and Management, Columbia University Mailman School of Public Health, New York, New York; 3 School of Social Work, Columbia University, New York, New York

## Abstract

This cross-sectional study compares health insurance coverage continuity, health care access, health care quality, and health care use between LGBTQ+ and non-LGBTQ+ individuals 12 to 14 months post partum.

## Introduction

Lesbian, gay, bisexual, transgender, queer, and other sexual and gender minority (LGBTQ+) people account for 18.1% of US parents but are often not identified in pregnancy and childbirth research.^1^ Structural discrimination may contribute to inequitable access to care and differential health care use between LGBTQ+ and non-LGBTQ+ postpartum people.^2^ We compared health insurance coverage continuity, health care access, care quality, and health care use between LGBTQ+ and non-LGBTQ+ individuals 12 to 14 months post partum.

## Methods

This cross-sectional study was approved by the institutional review boards at all study jurisdictions and Columbia University. We followed the STROBE reporting guidelines. Informed consent was obtained electronically or by phone. We used the 2020 Postpartum Assessment of Health Survey (PAHS), a follow-up survey to the 2020 CDC Pregnancy Risk Assessment Monitoring System, in 6 states (Kansas, Michigan, New Jersey, Pennsylvania, Utah, and Virginia) and New York City.^3,4,5^ The study sample included PAHS respondents with complete self-reported sexual orientation and gender identity data. We measured health insurance continuity, health care access, care quality, and health care use 12 to 14 months post partum.

We estimated unadjusted rates of each outcome stratified by LGBTQ+ status. We used age- and state-adjusted logistic regression models to estimate differences in probability of each outcome by LGBTQ+ status. We weighted all analyses to be representative of 2020 live births in the 7 jurisdictions.^4^ Two-sided *P* < .05 was considered statistically significant. Survey methods are detailed in the eMethods in Supplement 1.

## Results

The sample included 4427 postpartum people (mean [SD] age, 30.0 [5.6] years), of whom 5.1% (weighted) self-identified as LGBTQ+ (Table 1). In adjusted models (Table 2), LGBTQ+ people were significantly more likely to report delaying needed care (14.6 percentage points [PP]; 95% CI, 6.8-22.4 PP; *P* < .001), cost-related nonadherence (8.3 PP; 95% CI, 0.6-15.9 PP; *P* = .03), any primary care (11.8 PP; 95% CI, 1.6-21.9 PP; *P* = .02), any specialist care (15.3 PP; 95% CI, 5.3-25.4 PP; *P* = .003), any emergency department (ED) use (12.4 PP; 95% CI, 2.7-22.2 PP; *P* = .01), and low care quality (10.7 PP; 95% CI, 0.3-21.1 PP; *P* = .04) compared with non-LGBTQ+ people in the postpartum year. LGBTQ+ people were less likely to report any dental care (−14.4 PP; 95% CI, −24.7 to −4.1 PP; *P* = .006) compared with non-LGBTQ+ people. There were no significant differences in postpartum health insurance or health care visits.

**Table 1.** Participant Characteristics by LGBTQ+ Identity

Characteristic	Participants, No. (weighted %)^a^		*P* value^b^
	LGBTQ+ (n = 238)	Non-LGBTQ+ (n = 4189)	
Age group, y			
18-24	82 (27.3)	637 (18.4)	.049
25-29	69 (27.0)	1137 (25.7)	
30-34	58 (31.7)	1425 (33.3)	
≥35	29 (14.0)	990 (22.7)	
Race and ethnicity			
Asian, Native Hawaiian or Pacific Islander, Southwest Asian, Middle Eastern, or North African	14 (9.7)	307 (8.9)	.43
Black	37 (11.8)	538 (14.6)	
Hispanic or Latinx	25 (11.4)	668 (16.9)	
American Indian or Alaska Native	1 (0.2)	21 (0.3)	
White	133 (60.6)	2463 (54.9)	
Multiple races or ethnicities	28 (6.3)	190 (4.4)	
Educational level			
Less than high school graduate	20 (9.9)	267 (8.4)	.19
High school graduate	76 (28.3)	769 (21.2)	
More than high school graduate	142 (61.9)	3153 (70.4)	
Insurance at birth			
Commercial, military, or other	123 (51.6)	2748 (61.2)	.19
Medicaid	109 (45.6)	1324 (36.2)	
Uninsured	6 (2.8)	116 (2.6)	
Household income (percentage of the federal poverty level)			
≤138	110 (42.2)	1368 (35.2)	.43
139-199	29 (11.4)	415 (9.1)	
200-399	53 (23.9)	1258 (28.8)	
≥400	39 (19.2)	1028 (23.0)	
Missing	7 (3.1)	120 (3.9)	

Abbreviation: LGBTQ+, lesbian, gay, bisexual, transgender, queer, and other sexual and gender minority person.

^a^ Survey weights were designed to generate representative estimates of all live births in Kansas, Michigan, New Jersey, Pennsylvania, Utah, Virginia, and New York City. Missing observations less than 0.5% are not shown.

^b^ *P* values were estimated using χ^2^ tests comparing LGBTQ+ and non-LGBTQ+ respondents.

**Table 2.** Differences in Health Insurance Continuity, Health Care Access, and Health Care Use Between LGBTQ+ and Non-LGBTQ+ Postpartum Individuals

Outcome	LGBTQ+, weighted %	Non-LGBTQ+, weighted %	Unadjusted analysis		Adjusted analysis	
			Difference, weighted % (95% CI)	*P* value	Difference, % (95% CI)^a^	*P* value
Health insurance						
Health insurance continuity from childbirth to 12-14 mo post partum^b^						
Consistent coverage	88.2	88.8	−0.6 (−7.5 to 6.1)	.84	0.2 (−6.5 to 6.9)	.95
Insurance change	5.1	3.9	1.2 (−2.5 to 5.0)	.51	1.1 (−2.8 to 4.9)	.59
Insurance loss	5.4	5.4	−0.03 (−4.9 to 4.9)	.99	−0.02 (−4.9 to 4.5)	.93
Consistent uninsured	1.3	1.9	−0.6 (−2.6 to 1.5)	.61	−1.1 (−3.2 to 1.1)	.33
Uninsured any time since childbirth	15.2	11.6	3.6 (−4.8 to 12.1)	.40	3.0 (−5.8 to 11.7)	.51
Health care access and quality^c^						
No usual source of care	36.5	29.9	6.7 (−2.7 to 16.0)	.16	4.2 (−4.2 to 12.6)	.33
Delayed or did not get needed care	35.0	20.6	14.4 (6.7 to 22.0)	<.001	14.6 (6.8 to 22.4)	<.001
Cost-related nonadherence to prescription medication	12.7	4.2	8.5 (1.6 to 15.5)	.02	8.3 (0.6 to 15.9)	.03
Low health care quality rating (<8)	35.7	24.0	11.6 (8.4 to 22.4)	.04	10.7 (0.3 to 21.1)	.04
Health care use						
Any health care	87.4	87.5	−0.2 (−7.2 to 3.9)	.96	1.4 (−5.6 to 8.3)	.70
Attended postpartum visit	78.6	78.1	0.5 (−6.8 to 7.9)	.88	2.2 (−4.9 to 9.2)	.55
Lactation consultant	23.3	17.5	5.8 (−3.7 to 15.4)	.23	6.3 (−2.9 to 15.6)	.18
Any primary care^d^	62.1	51.2	10.9 (1.0 to 20.8)	.03	11.8 (1.6 to 21.9)	.02
Any specialist care^e^	33.8	19.4	14.4 (4.8 to 23.9)	<.001	15.3 (5.3 to 25.4)	.003
Any dental care	26.2	42.1	−15.9 (−24.3 to −7.5)	<.001	−14.4 (−24.7 to −4.1)	.006
Any emergency department use	29.7	16.6	13.1 (3.7 to 22.6)	.01	12.4 (2.7 to 22.2)	.01

Abbreviation: LGBTQ+, lesbian, gay, bisexual, transgender, queer, and other sexual and gender minority.

^a^ Adjusted differences in predicted probabilities estimated using a logistic regression model adjusting for respondent age group and jurisdiction.

^b^ Health insurance continuity measures whether insurance type at the time of childbirth was the same at 12 to 14 months post partum (consistent coverage) or whether the individual had a different insurance type (insurance change), was insured at childbirth but uninsured at 12 to 14 months post partum (insurance loss), or uninsured at both time points (consistent uninsured).

^c^ All health care access and quality measures except “usual source of care” are in reference to the timeframe “since giving birth” (eMethods in Supplement 1).

^d^ Any primary care includes care from a primary care physician, nurse, or physician assistant.

^e^ Specialist care includes psychiatrists, cardiologists, endocrinologists, orthopedists, urologists, surgeons, substance use or addiction treatment clinicians, and physical or occupational therapists. Specialist care does not include obstetricians-gynecologists.

## Discussion

Despite similar access to health insurance, LGBTQ+ people had large inequities in unmet health care needs and cost-related medication nonadherence in the postpartum year. Results suggested LGBTQ+ people receive lower-quality care in the postpartum period. Although use of pregnancy-related care was comparable, LGBTQ+ individuals used more primary and specialist care and were nearly twice as likely to use the ED compared with non-LGBTQ+ people.

Inequities in forgone care and medication nonadherence are consistent with prior literature documenting higher health care needs (eg, higher postpartum depression rates^6^), more financial barriers,^6^ and negative health care experiences among LGBTQ+ people.^3^ Differences in ED and specialty care use may reflect delayed or unmet health care needs, worse birth outcomes, and increased risk of postpartum mental health symptoms among LGBTQ+ people.^3,6^ We could not evaluate specific mechanisms because information on frequency of specialty care visits or services received was unavailable. Multilevel approaches to mitigating LGBTQ+-related inequities in access to and quality of care in the postpartum period include policies that institutionalize LGBTQ+ equality, clinician training for LGBTQ+-inclusive care, and clinician understanding of unique experiences of LGBTQ+ childbearing people. Study limitations include self-reported health care use and small LGBTQ+ sample size. Study participants, who gave birth in 2020, experienced the postpartum year during the peak years of COVID-19. Experiences of unique pandemic-related policies and care disruptions may not generalize to the postpandemic period.

## Notes

Supplement 1. **eMethods.** Study Data Sources eTable. Study Variable Sources and Definitions

Supplement 2.
Data Sharing Statement

## References

[1] Wilson BD, Bouton LJ. LGBTQ parenting in the US. Williams Institute, University of California, Los Angeles School of Law. 2024. Accessed March 13, 2025. https://williamsinstitute.law.ucla.edu/wp-content/uploads/LGBTQ-Parenting-Jul-2024.pdf

[2] Lacombe-Duncan A, Andalibi N, Roosevelt L, Weinstein-Levey E. Minority stress theory applied to conception, pregnancy, and pregnancy loss: a qualitative study examining LGBTQ+ people’s experiences. PLoS One. 2022;17(7):e0271945. doi:10.1371/journal.pone.0271945 35881607 PMC9321415

[3] Liu C, Underhill K, Aubey JJ, Samari G, Allen HL, Daw JR. Disparities in mistreatment during childbirth. JAMA Netw Open. 2024;7(4):e244873. doi:10.1001/jamanetworkopen.2024.4873 38573636 PMC11192180

[4] Daw JR, Underhill K, Liu C, Allen HL. The health and social needs of Medicaid beneficiaries in the postpartum year: evidence from a multistate survey. Health Aff (Millwood). 2023;42(11):1575-1585. doi:10.1377/hlthaff.2023.00541 37931190

[5] Daw JR, Allen HL, Underhill K. Postpartum Assessment of Health Survey (PAHS) 2020 technical documentation. 2023. Accessed August 13, 2024. https://www.publichealth.columbia.edu/file/15116/download?token=h3OrI__u

[6] Miller ML, Dupree J, Monette MA, Lau EK, Peipert A. Health equity and perinatal mental health. Curr Psychiatry Rep. 2024;26(9):460-469. 39008146 10.1007/s11920-024-01521-4

