# Supplementary material for: Postpartum Care Differences in LGBTQ+ and Non-LGBTQ+ Individuals
Source: JAMA Health Forum. 2025 May 2;6(5):e250672. doi: 10.1001/jamahealthforum.2025.0672 (PMC12048847; doi:10.1001/jamahealthforum.2025.0672)
Supplement: Supplement 1. — eMethods. Study Data Sources eTable. Study Variable Sources and Definitions [file jamahealthforum-e250672-s001.pdf]

# Supplemental Online Content

Nguyen KH, Daw JR, Allen HL. Postpartum care differences in LGBTQ+ and non-LGBTQ+ individuals. *JAMA Health Forum*. 2025;6(5):e250672. doi:10.1001/jamahealthforum.2025.0672:

**eMethods.** Study Data Sources

**eTable.** Study Variable Sources and Definitions

This supplemental material has been provided by the authors to give readers additional information about their work.

© 2025 Nguyen KH et al. *JAMA Health Forum*.

**eMethods** Study Data Sources

*Survey Overview:* The Postpartum Assessment of Health Survey (PAHS) is a follow-up survey to the CDC Pregnancy Risk Assessment Monitoring System (PRAMS). The 2020 PAHS included six states (KS, MI, NJ, PA, UT, VA) and New York City. PAHS jurisdictions were selected for having a large PRAMS sample size, consistently meeting the CDC PRAMS response rate thresholds, and willingness and capacity to collaborate with the research team. The 2020 PAHS sampling frame included all 2020 PRAMS respondents (i.e. those who had a live birth in 2020 and were randomly sampled for PRAMS in the participating jurisdictions) who opted-in (MI) or did not opt-out (other 6 jurisdictions) of being recontacted for PAHS. From January 2021 to March 2022, people in the PAHS sampling frame were contacted in monthly batches by phone, email and mail from 12-14 months after their live birth with the opportunity to participate in PAHS. Participants were required to provide verbal or written consent to participation and a $45 gift card was provided for survey completion.

*Measures:* The 2020 PAHS questionnaire contained 108 core questions about health and well-being; social needs; and health care in the first year postpartum, largely using validated question designs from other population health surveys. PAHS was offered in both English and Spanish. PAHS responses were linked on an individual-level to birth certificate variables and PRAMS survey responses. Table A1 shows the data source and definition for LGBTQ+ identity, each covariate and outcome variable.

*Sample Size and Response Rate:* The total 2020 PRAMS sampling frame across the 7 sites was N=14,314. Across the 7 sites, 28.9% of PRAMS respondents chose not to be recontacted for PAHS (19% in 6 opt-out sites; 78.9% in opt-in site of Michigan). This resulted in a total PAHS sampling frame of N=6021. Based only on complete responses, the unweighted PAHS response rate relative to the PAHS sampling frame was 76.4% (N=4598). The analysis for this study included only PAHS respondents with complete data on sexual orientation and gender identity, N=4427 (96.3%)

*Survey Weights:* The 2020 PAHS survey weights are designed to generate representative estimates of all live births in 2020 in the 7 participating jurisdictions. The PAHS weights build on the PRAMS survey weights and account for the stratified survey design of PRAMS as well as PRAMS and PAHS nonresponse. The weights are further calibrated on key characteristics including PRAMS sampling strata, maternal age, marital status, race, Hispanic ethnicity, education, and infant birthweight to align with total population values for all live births in the participating jurisdictions.

© 2025 Nguyen KH et al. *JAMA Health Forum*.

**eTable.** Study Variable Sources and Definitions

| Variable | Survey Question(s) | Measure Definition |
|---|---|---|
| **LGBTQ+** | *How do you describe your gender?*<br>*What do you consider your sexual orientation to be?* | 1 = [Gender = Trans Male/Trans Man OR Trans Female/Trans Women OR Genderqueer/ Gender Nonconforming] OR [Sexual Orientation = Gay OR Lesbian OR Bisexual OR Other self-described non-heterosexual (e.g. demisexual, asexual, queer)]<br><br>0 = [Gender = Female AND Sexual Orientation = Heterosexual or straight] |
| **Health Insurance** | | |
| Health Insurance Continuity from Childbirth to One Year Postpartum | *What kind of health insurance did you have when you gave birth?*<br><br>*What kind of health insurance do you have now?* | Survey responses categorized as Private, Medicaid, or Uninsured<br><br>Consistent Coverage= Medicaid at both time points or Private at both time points<br><br>Insurance Change= Medicaid at childbirth and Private at 12-14 months postpartum, or vice versa<br><br>Insurance Loss= Medicaid or Private at childbirth and Uninsured at 12-14 months pp<br><br>Consistent Uninsured= Uninsured at childbirth and 12-14 months pp |
| Uninsured Any Time Since Childbirth | *Since giving birth, have you ever been uninsured?* | 1 = Yes<br>0 = No |
| **Health Care Access and Quality** | | |
| No Usual Source of Care | *Where do you USUALLY go if you are sick and need health care?* | 1 = “I don’t have a usual place” OR “A hospital emergency room” OR “An urgent care clinic” |

© 2025 Nguyen KH et al. *JAMA Health Forum*.

| | | |
|---|---|---|
| | | 0 = “A private doctor’s office or clinic” OR “A public health clinic, community health center, or tribal clinic” OR “Some other place not listed here” |
| Delayed/Did Not Get Needed Care | *Since giving birth, was there ever a time when you needed health care but you DELAYED getting care or DID NOT GET the care you needed?* | 1 = Yes<br>0 = No |
| Cost-Related Nonadherence | *Not including over the counter medications, since giving birth was there ever a time when you did not fill a prescription or take your medication as prescribed because of COST? This includes skipping or splitting doses to make your medication last longer.* | 1 = Yes<br>0 = No |
| Low Health Care Quality Rating PP (<8) | *Using a number from 0 to 10, where 0 is the worst health care possible and 10 is the best health care possible, what number would you use to rate your health care in general since giving birth?* | <8 = Yes<br>>=8 = No<br><br>The mean score (8) was selected as a cut-off. |
| **Health Care Use** | | |
| Any health care | *Have you received any health care since giving birth? This includes any health care for your physical, mental, or dental needs.* | 1 = Yes<br>0 = No |
| Attended Postpartum Visit | *In the first two months after you gave birth, did you have a postpartum checkup for yourself with a health care clinician such as a doctor, nurse, or midwife? Include checkups that you had over the phone or online. Don’t include labor and delivery care.* | 1 = Yes<br>0 = No |
| Lactation Consultant | *Since giving birth, aside from the physicians/doctors noted above, have you seen any of the following other types of health care providers?* | 1 = Selected “Lactation consultant”<br>0 = Did not select “Lactation consultant” |
| Any Primary Care (Physicians/Nurse/Physician Assistants Only) | *Since giving birth, have you seen any of the following types of physicians/doctors? Include visits that you had over the phone or on videoconference.*<br><br>*Since giving birth, aside from the* | 1 = “Primary care physician, family physician, or internist” OR “Nurse practitioner or physician assistant” |

© 2025 Nguyen KH et al. *JAMA Health Forum*.

| | | |
|---|---|---|
| | *physicians/doctors noted above, have you seen any of the following other types of health care providers?* | 0 = Did not select any of the<br>above providers |
| Any Specialist Care | *Since giving birth, have you seen any of the following types of physicians/doctors? Include visits that you had over the phone or on videoconference.*<br><br>*Since giving birth, aside from the physicians/doctors noted above, have you seen any of the following other types of health care providers?* | 1 = "Psychiatrist" OR<br>"Cardiologist (e.g., doctor for heart conditions)" OR<br>"Endocrinologist (e.g., doctor for diabetes or thyroid)" OR<br>"Orthopedist (e.g., doctor for muscles and bones)" OR<br>"Urologist (e.g., doctor for bladder)" OR<br>"Surgeon" OR<br>"Substance use or addiction treatment provider" OR<br>"Physical or occupational therapist" |
| Any Dental Care | *Since giving birth, aside from the physicians/doctors noted above, have you seen any of the following other types of health care providers?* | 1 = Selected "Dentist"<br>0 = Did not select "Dentist" |
| Any Emergency Department Use | *Since giving birth, how many times did you go to an emergency room to get care for yourself?* | 1 = One or more times<br>0 = Never |

© 2025 Nguyen KH et al. *JAMA Health Forum*.
