# Supplementary material for: Postpartum Care Differences in LGBTQ+ and Non-LGBTQ+ Individuals
Source: JAMA Health Forum. 2025 May 2;6(5):e250672. doi: 10.1001/jamahealthforum.2025.0672 (PMC12048847; doi:10.1001/jamahealthforum.2025.0672)
Supplement: Supplement 2. — Data Sharing Statement [file jamahealthforum-e250672-s002.pdf]

Data Sharing Statement

Nguyen. Postpartum Care Differences in LGBTQ+ and Non-LGBTQ+ Individuals. *JAMA Health Forum*. Published May 02, 2025. doi:10.1001/jamahealthforum.2025.0672

Data
Data available: No
